# Involvement of microRNA/cystine/glutamate transporter in cold-stressed gastric mucosa injury

**DOI:** 10.3389/fphar.2022.968098

**Published:** 2022-09-28

**Authors:** You-Cong Yin, Xiao-hui Li, Xuan Rao, Yuan-Jian Li, Jie Du

**Affiliations:** ^1^ Department of Pharmacology, School of Pharmaceutical Sciences, Central South University, Changsha, China; ^2^ Department of Pharmacy, The Central Hospital of Shaoyang, Shaoyang, China; ^3^ Department of Pharmacy, Xiangya Hospital, Central South University, Changsha, China; ^4^ National Clinical Research Center for Geriatric Disorders (XIANGYA), Central South University, Changsha, China

**Keywords:** microRNA, stress ulcer, cold stress, l-glutamate, cystine/glutamate transporter, apoptosis

## Abstract

Stress ulcers are complicated by severe trauma and other critical diseases, the mechanism of which remains unclear. An increasing number of studies have shown that microRNAs (miRNAs) are important regulators of stress responses such as hypoxia, abnormal temperature, and inflammation. The evidence indicates that miRNAs are also involved in regulating stress-induced ulcers. Recently, we demonstrated that gastric mucosal injury induced by aspirin is related to the reduction of glutamate levels by inhibition of cystine/glutamate transporter (xCT) activity. In the present study, the effect of a miRNA/xCT on gastric mucosal injury induced by cold stimulation was investigated. We found that cold stimulation induced gastric mucosa injury with a reduction in glutamate levels and xCT activity and upregulation of miR-143, miR-152, and miR-181 expression. Exogenous glutamate significantly alleviated gastric mucosa injury by cold stimulation. *In vitro* experiments demonstrated that treatment with miR-143, miR-152, or miR-181 mimics directly induced cell damage. The effects of these mimics were alleviated by exogenous glutamate. The present study suggests that miR-143, miR-152, and miR-181 are involved in cold stimulation-induced acute gastric mucosal injury. Furthermore, the regulatory effect of miRNAs on gastric mucosa injury induced by cold stimulation is related to a decrease in glutamate release by reduction of cystine/glutamate transporter activity.

## Introduction

Stress response refers to the non-specific systemic reaction that occurs when the body is stimulated by various strong factors, including severe trauma, burns, and septic shock, that may present with symptoms and multiple organ dysfunction (Brzozowska et al., 2014; Gyires et al., 2015). Stress stimulates the sympathetic nervous system of hypothalamus resulting in pituitary-adrenal axis disorders, promoting shrink blood vessels, promote oxidative stress and inflammation (Ginsberg et al., 2010). Stress-related disorders involve multiple systems of the body and are associated with subsequent diseases, such as neuroendocrine disorders, immune diseases, and stress ulcer diseases. Among them, acute stress-related gastric lesions, which manifest as acute gastrointestinal mucous erosion and even bleeding, are clinical concerns (Bhatia and Tandon, 2005). Clinical data show that the incidence of stress ulcers is as high as 15%–50% in critically ill patients who have suffered severe disease events (Valledor et al., 2013). As the pathogenesis of stress ulcers is complex and remains unclear, agents for inhibition of acid secretion and gastric mucous protection are mainly used in clinical symptomatic treatment. The gastric injury involves an imbalance in mucous defense and injury. Gastric ulcers induced by *Helicobacter pylori* infection and nonsteroidal anti-inflammatory drugs (NSAIDS) are related to the increase in some inflammatory factors (Wang et al., 2020), cytokines, and reactive oxygen species (Wang et al., 2021), which can impair gastric mucous (Ren et al., 2021). Further, these gastric ulcers can also contribute to decreased secretion of endogenous substances such as nitric oxide (Dejban et al., 2020), prostaglandins (Wang et al., 2021), and glutamate (Akhigbe et al., 2022), which have protective effects on the stomach. For example, glutathione significantly decreases the extent of ethanol-induced macroscopic injury to the mucosa of the gastric body and antrum (Loguercio et al., 1993). Our previous studies reported that exogenous glutamate has a protective effect against gastric mucosal injury induced by acute aspirin irritation (Du et al., 2014) and *H. pylori* infection (Du et al., 2020). Whether the reduction in endogenous active substances is also associated with stress-induced gastric injury remains unclear.

Recent studies have shown that glutamate plays an important role in the protection and repair of gastric mucous injuries (Du et al., 2014; Du et al., 2020; Xu et al., 2021). Glutamate, an important endogenous active substance, is widely present in the central nervous system and peripheral tissues. Physiological concentrations of glutamate are necessary to maintain cell function. Abnormal glutamate metabolism can lead to a series of diseases. Previous studies have focused on the cytotoxic effects of excessive glutamate in the lung, kidney, cardiac muscle tissue, and nervous system (Lau and Tymianski, 2010; Hamasato et al., 2013; Du et al., 2016). The protective effect of glutamate against gastric mucosal injury has been confirmed. Exogenous glutamate can also repair injuries induced by cold stimulation (Chen et al., 2001). The alteration in endogenous glutamate transportation may likely result in gastric mucosal injury induced by multiple factors. Therefore, we speculate that the glutamate pathway may also play an important role in stress-induced gastric ulcers, especially in apotosis process through value the balance of pro-apoptotic protein (Bax) and apoptosis suppressor protein (Bcl-2).

The extracellular level of glutamate is critically controlled by the cystine/glutamate transporter (xCT). The light chain xCT of this system is functionally important in the regulation of gastric function. A reduction in xCT activity in aspirin- and *H. pylori*-induced ulcer models has been demonstrated. Some stress factors reportedly affect xCT activity. For example, an activated integrated stress response induced by salubrinal promotes cisplatin resistance in human gastric cancer cells *via* enhanced xCT expression (Wang et al., 2018). Another study showed that stress-induced inhibition of nonsense RNA regulates intracellular cystine transport through the regulation of xCT (Martin and Gardner, 2015). Based on these findings, we speculated that stress ulcers may also be related to xCT. Cold stress is often used to induce a gastric ulcer model, hence, we intend to study that gastric mucosal injury induced by cold stress may also be related to the activity of glutamate transporters.

MicroRNAs (miRNAs) regulate gene transcription and are involved in the regulation of cell proliferation, differentiation, apoptosis, and immunity. Important roles of miRNAs in the stress response have been demonstrated. Various stress factors, including high temperature, cold, ischemia, stress, infection, and trauma, can lead to miRNA profile changes (Biggar and Storey, 2015). Exposure to cold is a direct threat to the body and induces changes in miRNA expression. For example, the expression of miR-16 and miR-21 in the muscle and liver are upregulated during hibernation (Biggar and Storey, 2015). The expression of miR-151, miR-425, miR-98, miR-328a, and miR-210 in the liver are significantly increased during cold stress (Lyons et al., 2015). Because the expression of some miRNAs has been closely related to gastric mucosa damage, we speculated that cold stress may induce upregulation of certain miRNAs to selectively inhibit the xCT target gene, resulting in a reduction of glutamate transport activity. In the present study, we explored the role of miRNA/xCT/glutamate pathway in cold stress-related ulcers.

## Materials and methods

### Animal and cell experiments

Experiments were performed using male Sprague–Dawley rats weighing 250–300 g. Our study was carried out in accordance with the Declaration of Helsinki and/or the Guide for the Care and Use of Laboratory Animals as adopted and promulgated by the National Institutes of Health. The rats were randomly divided into two groups (*n* = 8 per group): control, cold stress. After the cold stress model was successfully established, other rats were randomly divided into four groups (*n* = 8 per group): control, cold stress, cold stress + low-dose glutamate (4 mg/kg), and cold stress + high-dose glutamate (8 mg/kg) groups. The rats were housed in the same indoor environment at a temperature of 25°C and were fasted for 24 h before the establishment of the model, raised separately, and allowed to drink water freely. The control group and the cold stress group was given 0.9%NS (The same volume as the treatment group) gavage continuously for 3 days at the same time every day. In the cold stress group, after given gavage continuously for 3 days, on the third day, half an hour after gavage, the rats were positioned in a cylindrical rat retainer at 4°C for 3.5 h. Glutamate group was given glutamate (4 mg/kg or 8 mg/kg) gavage continuously for 3 days, in the same way, the rats were positioned in a cylindrical rat retainer at 4°C for 3.5 h then all the rats were sacrificed under anesthesia with 10% chloral hydrate (0.3 ml/100 g), and gastric juice was collected. The glutamate concentration in gastric juice was determined using a commercial kit provided by Nanjing Jiancheng Bioengineering Institute (www.njjcbio.com, product number: A074-1-1). Principle is as follows: glutamate + NAD++H2O→α-Ketoglutaric acid + NADH + NH4+, One hundred microliters of each sample was added to the substrate solution and the absorbance at 340 nm was measured using a spectrophotometer. The gastric tissues of all rats were excised by cutting along the greater curvature of the stomach. The tissues were washed and photographed. Gastric ulcers were measured under a dissecting microscope with a square grid micrometer. The number of ulcer lesions and the ulcer lengths in the glandular portion of the stomach were calculated and expressed as the ulcer index (UI). The gastric mucosal index was calculated using the GUTH method (Chang et al., 2007). GUTH method: The sum of the length of each lesion in the whole stomach was the injury Index, which was expressed as mm. Damage ≤1 mm (including erosion point) is 1 point; l mm < Damage ≤2 mm is 2 points; 2 mm < Damage ≤3 mm is 3 points; 3 mm < Damage ≤4 mm is 4 points; > 4 mm is 5 points; Damage width > 2 mm is Double UI. and the area of the lesions was summed up. One portion of the gastric tissue was fixed in 4% paraformaldehyde, embedded in paraffin, and cut into 5-μm sections. Some slices were stained with hematoxylin and eosin for morphometric analysis. The remaining gastric mucosal tissue was used for subsequent experiments. Cell death by apoptosis in the gastric tissue was evaluated by terminal deoxynucleotidyl transferase dUTP nick end labeling (TUNEL) staining and determination of caspase-3 activity.

A human gastric epithelial immortalized cell line (GES-1) was cultured at 37°C under 5% CO_2_ in Dulbecco’s modified Eagle medium containing 10% fetal bovine serum. After overnight incubation, the medium was replaced with a fresh medium. Cells were treated with miRNA mimics and L-glutamate (10^−5^–10^−6^ M) in a dose-dependent manner. Hoechst staining, caspase-3 assay, and TUNEL staining were used to evaluate apoptosis. Cell viability was measured using a lactate dehydrogenase assay.

### Collecting gastric secretion

Animals were anesthetized with 10% chloral hydrate (0.3 ml/100 g), and the pylorus was ligated in the above groups. After 30 min, the gastric cardia was ligated and the whole stomach was removed. The gastric juice was extracted with a syringe, centrifuged at 1500 rpm for 15 min, and the supernatant was frozen and stored.

### Immunohistochemistry

Tissue sections were placed in a water bath at 80°C for 20 min with antigen repair solution (citric acid 0.4 g, trisodium citric acid 3 g) in 1,000 ml distilled deionized water. After natural cooling, the sections were soaked in PBS for 5 min twice, followed by soaking in 3% hydrogen peroxide (H_2_O_2_) for 5 min and two 5-min changes of PBS. The sections were incubated with a blocking solution of primary antibody diluent and PBS in a 1:1 ratio at indoor environment at a temperature of 25°C for 1 h. The slices were incubated with primary antibody (1:50) overnight at 4°C. The corresponding secondary antibody (1:300) was then added and incubated at indoor environment at a temperature of 25°C for 1 h. 3,3′-Diaminobenzidine (DAB) was added to permit color development.

### miRNA mimic transfection experiment

Cells were plated and allowed to grow to 80%–90% confluency. They were released from the substrate using 0.25% trypsin and inoculated into cell culture plates for transfection experiments. miRNA mimic 20 μM reserve solution was added (2.5 µL) to 50 μL of 1× riboFECTTMCP buffer to prepare liquid A. Six microliters of 1× riboFECTTMCP reagent were added to 50 μL of 1× riboFECTTMCP buffer to prepare liquid B. Liquid A and liquid B were mixed at room temperature and allowed to stand for 15 min. The mixture was added to the culture plate, followed by the addition of 891.5 μL of culture base, and then placed in a cell culture box for transfection for 24 h.

### miRNA *in situ* hybridization experiment

The experiment was performed using digoxin-labeled oligonucleotide probes specific to miR-143, miR-152, and miR-181. Paraffin sections were routinely dewaxed in water and then exposed to mRNA DNA fragments. The sections were post-fixed in 1% polyformaldehyde in 0.1 m PBS (pH 7.2–7.6) containing a 1:1,000 dilution of diethylpyrocarbonate (DEPC). prehybridization, hybridization and closed drops: 37°C for 30 min. Streptavidin biotin-peroxidase complex (SABC) and DAB were added for color development.

### Real-time PCR analysis

Real-time PCR was performed to quantify the mRNA levels of xCT and miRNAs. Total RNA was extracted from gastric tissues and GES-1 cells using TRIzol reagent (TaKaRa Bio, Dalian, China). Five hundred nanograms of RNA from each sample were subjected to reverse transcription using the PrimeScript reverse transcription reagent kit (TakaRa Bio). The concentration and purity of the eluted RNA were determined spectrophotometrically by the ratio of optical density at 260 nm/280 nm between 1.8 and 2.2. Quantitative analysis of mRNA expression was performed using an ABI 7300 real-time PCR system (Applied Biosystems, Foster City, CA, United States) with SYBR Premix Ex Taq™ (TaKaRa Bio). PCR cycling conditions were an initial incubation at 95°C for 15 s, followed by 40 cycles of denaturation at 95°C for 5 s and annealing at 60°C for 31 s. The generated cDNA was amplified by quantitative real-time PCR with specific forward and reverse primers for human-xCT (F:5′-gtggcagtgaccttttctgag-3′, R:5′-cccatacccaccatcacacc-3′), rat-xCT (F:5′-cctctgttcatcccagcatta-3′, R:5′-cccagtcaaggtgataaggaag-3′), human β-actin (F:5′-gcaccacaccttctacaatga-3′, R:5′-gtcatcttctcgcggttggc-3′), and rat-β-actin (F:5′-tgtcaccaactgggacgata-3′, R:5′-accctcatagatgggcacag-3′). Data analysis was performed by the comparative Ct method using the ABI software. β-Actin was used to normalize mRNA expression.

### SDS-PAGE and western blots

For western blotting, equal protein amounts (60 μg) were separated using 10% SDS-PAGE. Primary antibodies against xCT (1:1,000; Abcam, Cambridge, United Kingdom), BAX (1:500; Boster Biological Technology, Pleasanton, CA, United States), and BCL-2 (1:500; Boster Biological Technology) were used. Secondary antibodies were horseradish peroxidase (HRP)-anti-mouse (1:5000; Boster Biological Technology) and HRP-anti-rabbit (1:5000; Boster Biological Technology). The relative optical density of each band was densitometrically analyzed, and the results were expressed as the ratio normalized to glyceraldehyde 3-phosphate dehydrogenase (Beyotime Biotechnology, Shanghai, China).

### Statistical analyses

The results are presented as the mean ± standard deviation (SD). Statistical analysis was performed using ANOVA followed by the Newman–Student–Keuls test for multiple comparisons. Statistical significance was set at *p* < 0.05.

## Results

### Decreased xCT activity of gastric mucosa in cold-stressed rats

**FIGURE 1:**
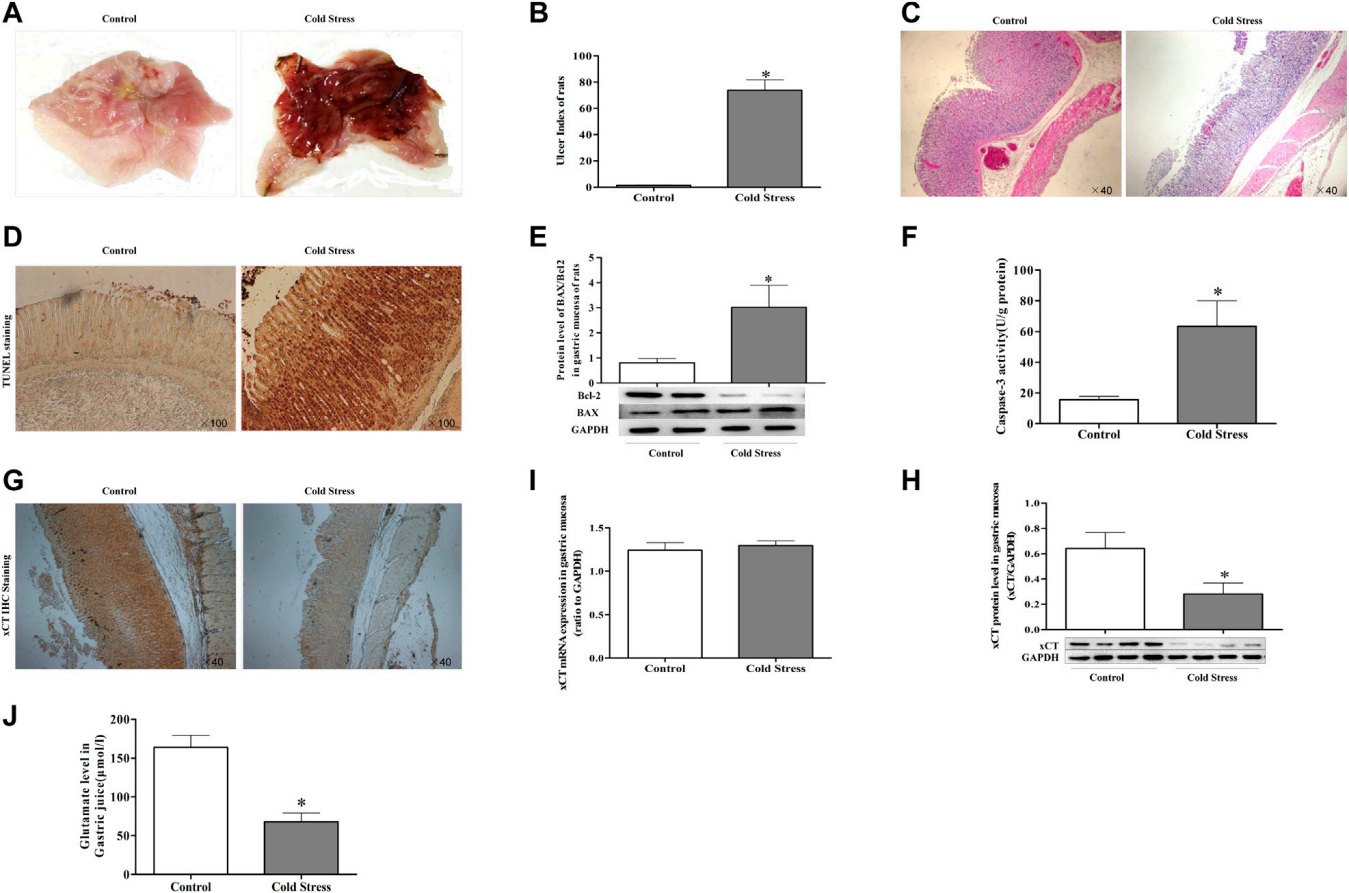
Decreased cystine/glutamate transporter (xCT) in gastric mucosa of cold-stressed rats. **(A)** gastric mucosa injury in rats. **(B)** Gastric mucosal ulcer index of rats. **(C)** Morphological changes in rat gastric tissue observed using light microscopy (×40). **(D)** TUNEL staining of gastric tissue as observed using light microscopy (×100). **(E)** The ratio of pro-apoptotic protein BAX to anti-apoptotic protein Bcl-2. **(F)** Activity of caspase-3 in gastric tissue. **(G)** xCT expression in gastric tissue (×40). **(H)** Protein expression of xCT. **(I)** xCT mRNA expression. **(J)** Glutamate content in gastric juice. Data are presented as mean ± standard error (*n* = 8). *p < 0.05 vs Control.

The stomach exhibited mucous swelling, hyperemia, and linear hemorrhages when rats were subjected to cold stimulation (Figure 1A). A tendency toward increased gastric ulcer index was evident (Figure 1B). The gastric epithelium structure was arranged loosely and was disordered, with evident shedding of cells accompanied by a large number of inflammatory cells (Figure 1C). In addition, in TUNEL staining, we could see that cold stress resulted in apoptosis of gastric mucosal cells. (Figure 1D). Assays of caspase-3 activity and expression of apoptosis-related proteins displayed similar results as with TUNEL staining (Figures 1E,F). These results revealed the obvious injury and apoptosis induced by cold stress. At the same time, we detected the expression of glutamate transporter. A lower level of xCT protein was detected in the cold stress group compared with that in the control (Figures 1G,H), while the expression of xCT mRNA was not significantly changed (Figure 1I). As xCT transports glutamate to the cell exterior, the concentration of glutamate in gastric juice was calculated (Figure 1J), it showed a decreased glutamate in cold-stressed rats. These results indicate that the decreased activity of xCT may contribute to ulcers caused by cold stress.

### Exogenous glutamate supplementation attenuates gastric mucosa injury induced by cold stress

**FIGURE 2:**
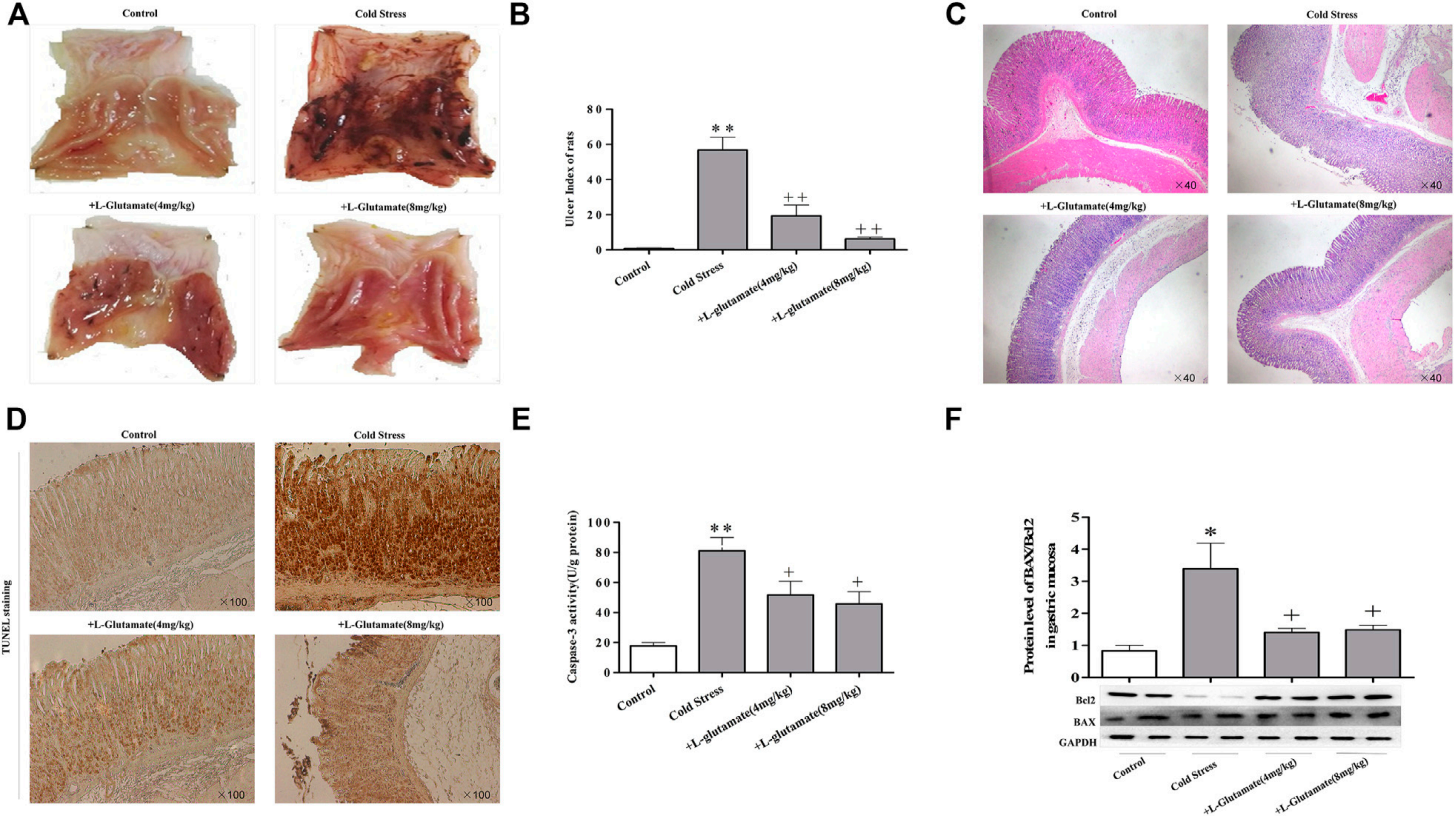
Exogenous glutamate supplementation attenuates gastric mucosa injury induced by cold stress. **(A)** Gastric mucosal injury in rats. **(B)** Gastric mucosal ulcer index of rats. **(C)** Morphological changes of rat gastric tissue by light microscopy (×40). **(D)** Apoptosis observed in sections (×100). **(E)** Caspase-3 activity. **(F)** Expression of BAX/Bcl-2. Data are expressed as mean ± standard error (*n* = 8). *p < 0.05, **p < 0.01 vs. Control, +*p* < 0.05, ++*p* < 0.01 vs. Cold stress. Glu: Glutamate.

To evaluate the effects of L-glutamate on cold-stressed gastric mucous ulceration, rats were pretreated with L-glutamate at doses of 4 or 8 mg/kg once a day for 3 days before cold restraint. The glutamate-supplemented group showed reduced gastric mucosal swelling, congestion, and epithelial shedding (Figure 2A), accompanied by a significant decrease in the ulcer index (Figure 2B). Morphologically, rats pretreated with L-glutamate displayed significantly attenuated gastric lesions. Dilation of gastric epithelial cells, loss of mucosal architecture, and exfoliation were not significant, as observed in the ulcer non-pretreated group (Figure 2C). Tunel staining showed that glutamate had a protective effect on cold stress-induced apoptosis (Figure 2D). Furthermore, glutamate pre-treatment reduced caspase-3 activity (Figure 2E), with a decreased ratio of the apoptotic protein BAX and the anti-apoptosis protein Bcl-2 (Figure 2F).

### Altered miRNA expression in rats under cold stress

**FIGURE 3:**
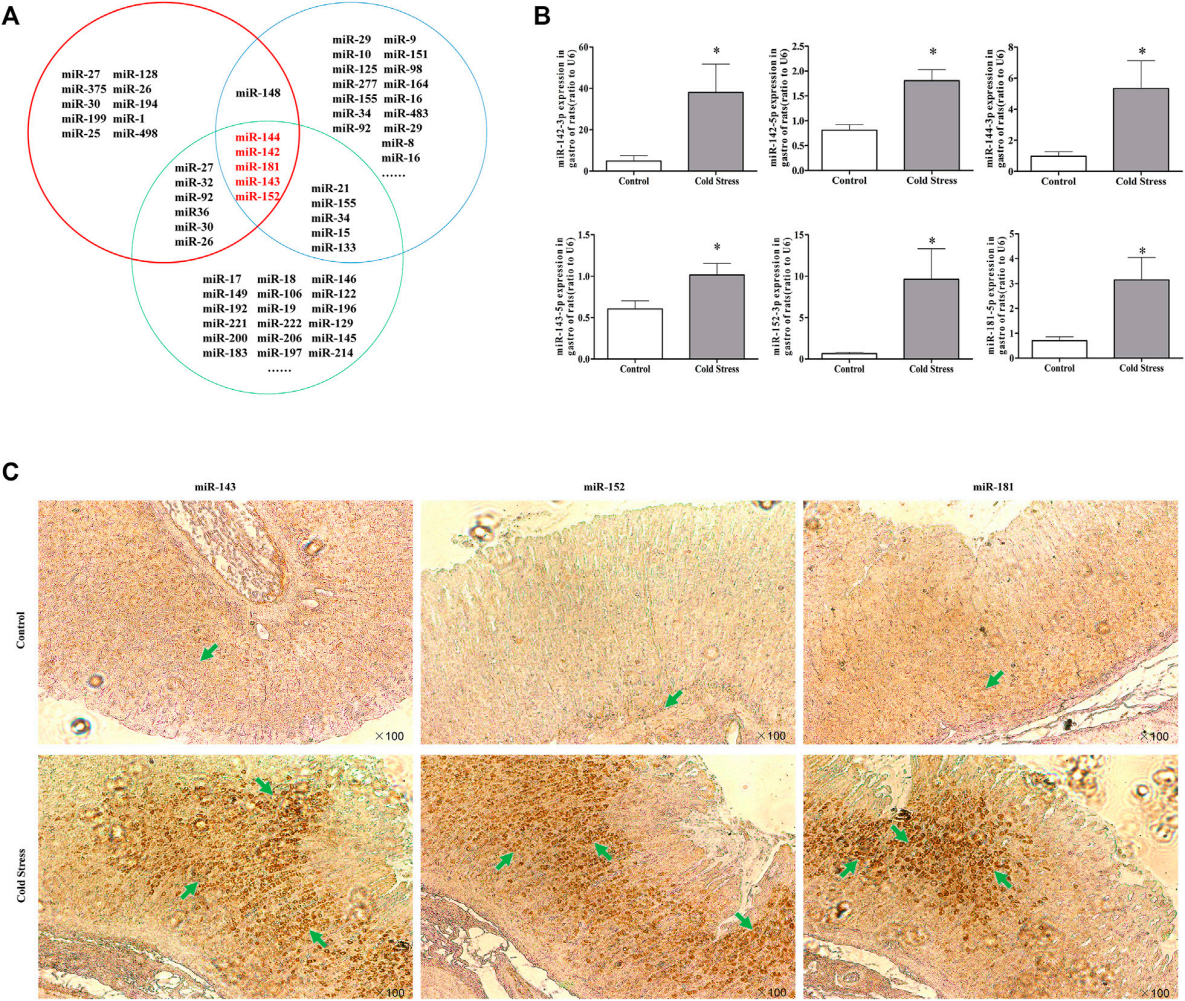
Change in miRNA expression in rats under cold stress. **(A)** Bioinformatics analysis of cold stress, apoptosis, and xCT-related miRNAs. The red circles represent xCT-related miRNAs; the green circles represent cold stress-related miRNAs; the blue circles represent apoptosis-related miRNAs. **(B)** Changes in miRNA expression in gastric tissues during cold stress. Data are expressed as mean ± standard error (*n* = 8). *p < 0.05 vs Control. **(C)** Effect of cold stress on miRNA expression in gastric tissues (100 *in situ* hybridization observations).

To explore the mechanisms responsible for xCT downregulation in cold-stressed rats, potential candidate miRNAs were screened using the miRDB Targets, miRanda, and TargetScan 6.2. Based on the bioinformatics results and our pilot study, miRNA-143, miRNA-152, and miRNA-181 were identified as potential xCT regulators (Figures 3A,B). Consistent with our real-time PCR findings, *in situ* hybridization revealed significantly increased expression of these miRNAs compared to expression in the control rats (Figure 3C). These results indicate a correlation between miRNAs and xCT during cold stress.

### Mimics of miRNAs suppress the expression of xCT and induce cell injury

**FIGURE 4:**
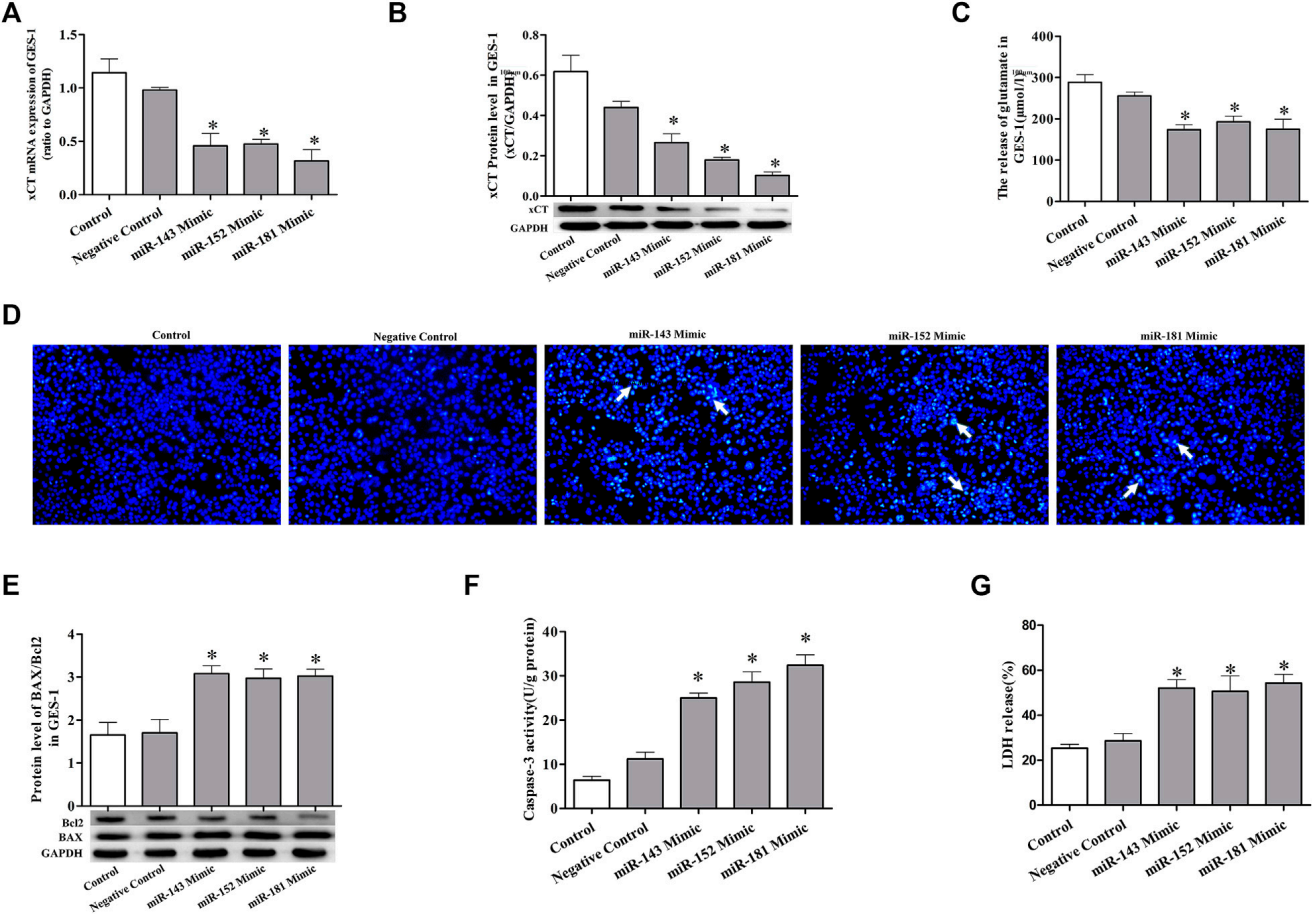
miRNA mimics suppress the expression of xCT and induce cell injury. **(A)** xCT mRNA level in GES-1 cells. **(B)** The protein expression level of xCT in GES-1 cells. **(C)** The concentration of released glutamate in GES-1 cells. **(D)** Detection of apoptosis (×100). **(E)** The ratio of pro-apoptotic protein BAX to anti-apoptotic protein Bcl-2 used to detect apoptosis. **(F)** Caspase-3 activity. **(G)** LDH release. Data are expressed as mean ± standard error (*n* = 3). *p < 0.05 vs. Negative Control.

To further confirm the regulatory effect of miRNAs on cold stress-induced ulcers, mimics were used. Transfection of GES-1 cells with miRNA-143, miRNA-152, and miRNA-181 mimics significantly decreased mature miRNA expression accompanied by xCT mRNA, and protein expression was significantly suppressed (Figures 4A,B). Reduced glutamate levels were also detected in the cell culture medium (Figure 4C). Examination of the effects of the three mimics on GES-1 cell apoptosis revealed that in the absence of cold stress, the mimics increased apoptotic compared to that in the negative control group (Figure 4D). We also observed upregulated levels of pro-apoptotic BAX protein and downregulated levels of apoptosis inhibitory protein Bcl-2 in the presence of the mimics compared with the control (Figure 4E), as well as higher caspase-3 activity in the mimic groups (Figure 4F). Lactate dehydrogenase (LDH) assays showed that the mimic groups featured significantly greater cell damage than the control group (Figure 4G). The latter finding implicated LDH as a potential biochemical marker of cell injury. The collective results suggest that miRNA mimics significantly promote apoptosis and damage.

### Cell damage induced by miRNA mimics can be attenuated by exogenous glutamate supplementation

**FIGURE 5:**
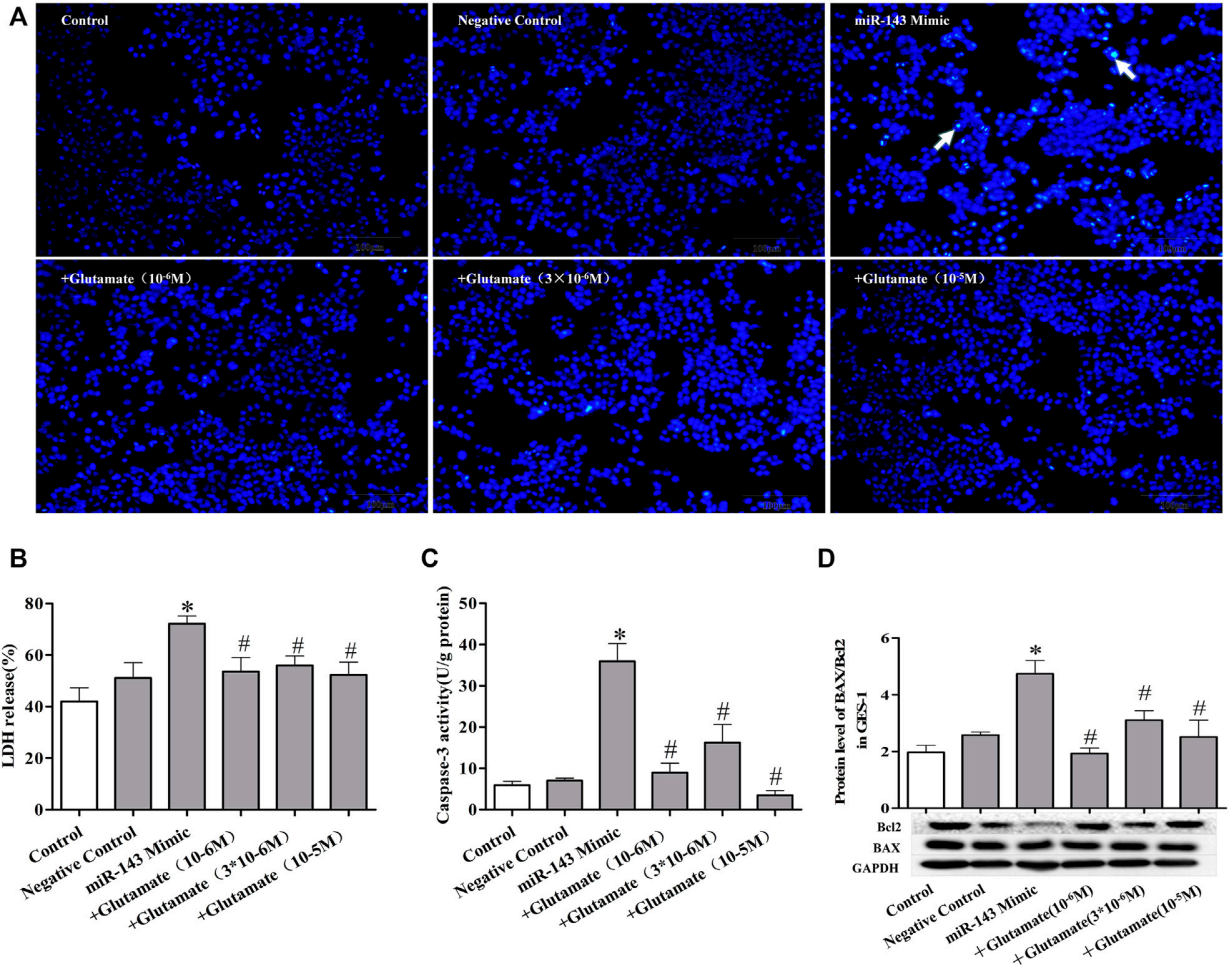
Cell damage induced by miRNA mimics can be attenuated by exogenous glutamate supplementation. **(A)** Protective effect of glutamate on gastric mucosal epithelial cell apoptosis after treatment with mimic miR-143 (Hoechst staining, ×100). **(B)** LDH release. **(C)** Caspase-3 activity. **(D)** The ratio of BAX/Bcl-2 in gastric mucosal epithelial cells. Data are expressed as mean ± standard error (*n* = 3). #*p* < 0.05 vs. MicroRNAs mimic, *p < 0.05 vs. Negative Control.

**FIGURE 6:**
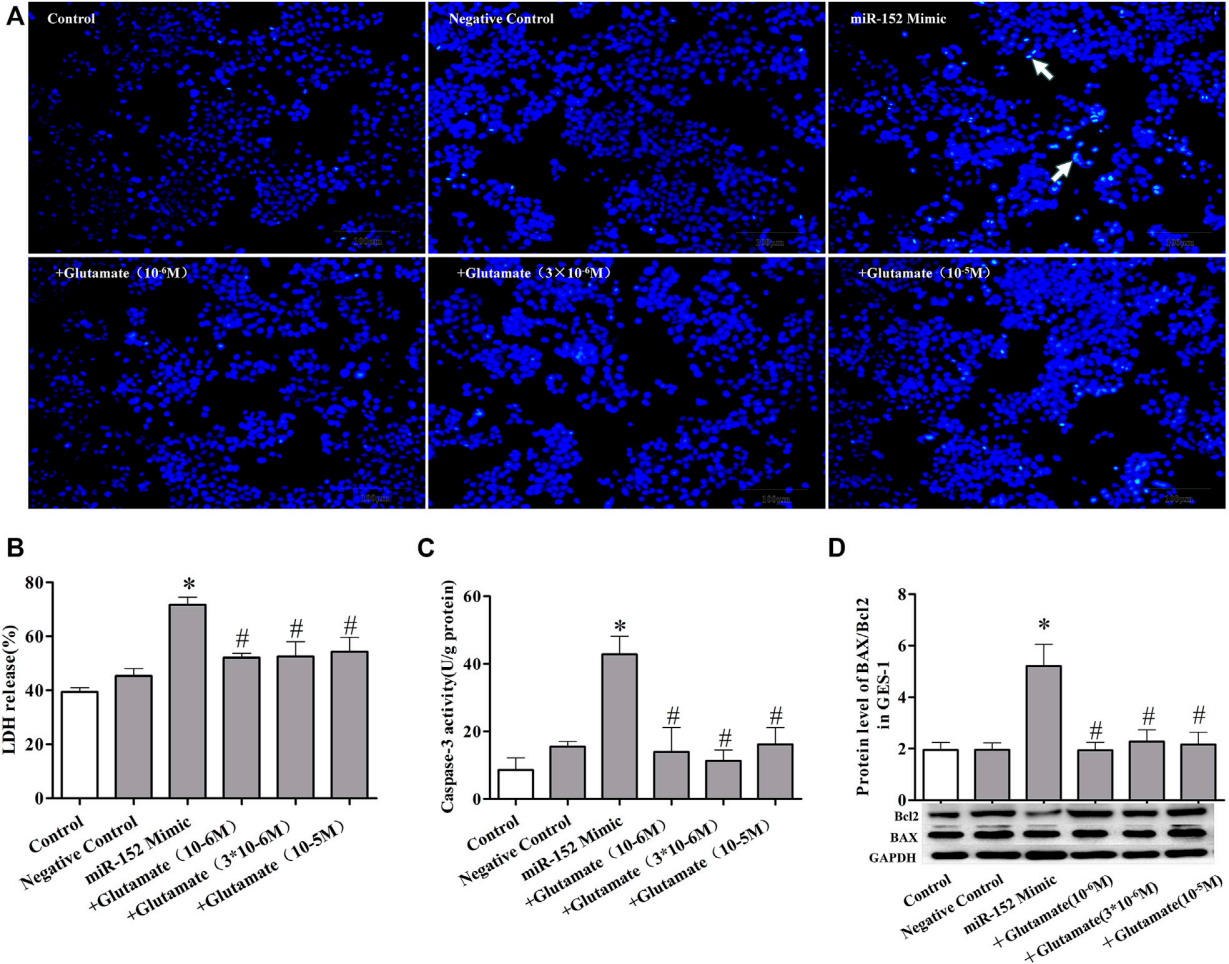
Cell damage induced by miRNA mimics can be attenuated by exogenous glutamate supplementation. **(A)** Protective effect of glutamate on gastric mucosal epithelial cell apoptosis after treatment with mimic miR-152 (Hoechst staining, ×100). **(B)** LDH release. **(C)** Caspase-3 activity. **(D)** The ratio of BAX/Bcl-2 in gastric mucosal epithelial cells. Data are expressed as mean ± standard error (*n* = 3). #*p* < 0.05 vs. MicroRNAs mimic, *p < 0.05 vs. Negative Control.

**FIGURE 7:**
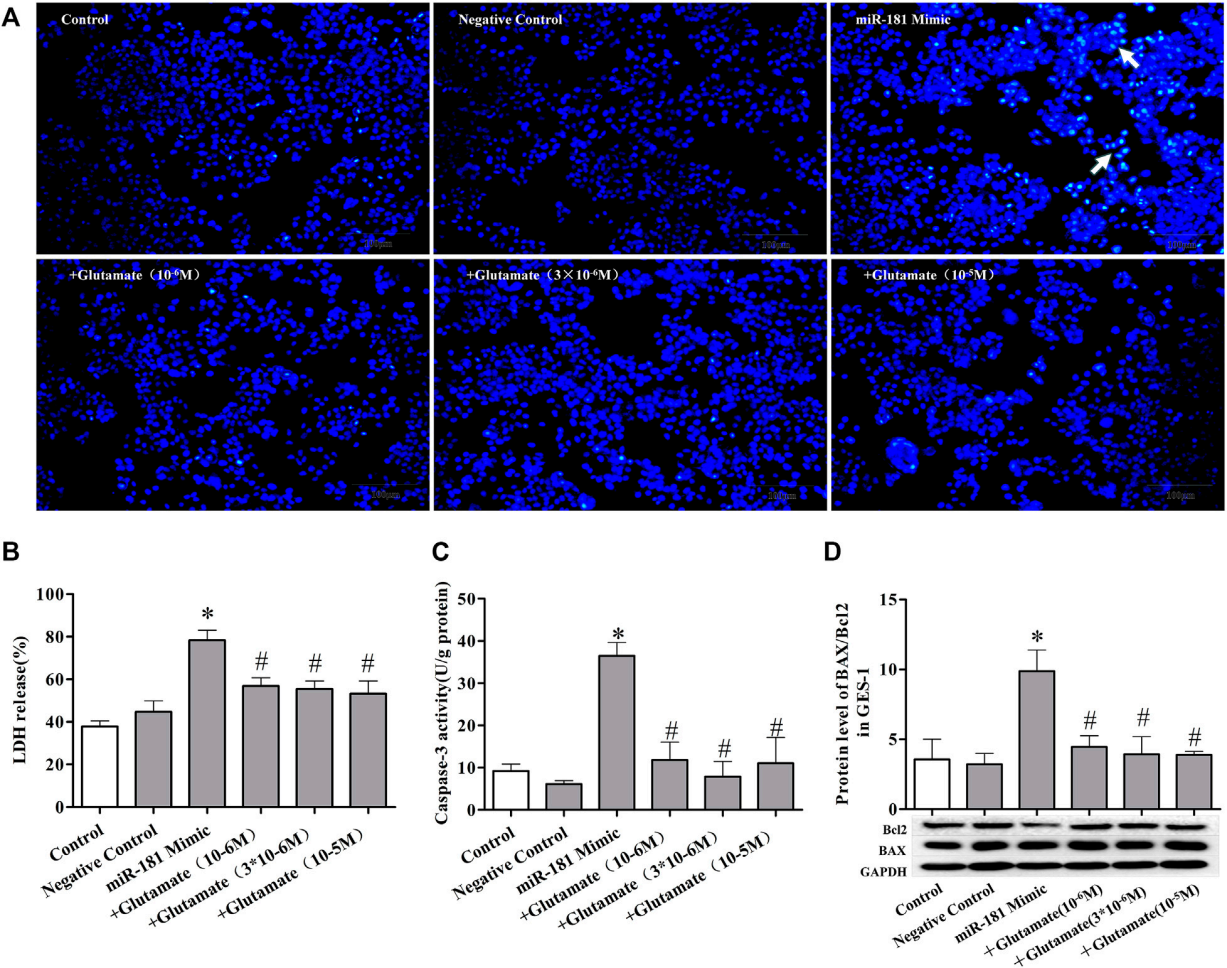
Cell damage induced by miRNA mimics can be attenuated by exogenous glutamate supplementation. **(A)** Protective effect of glutamate on gastric mucosal epithelial cell apoptosis after treatment with mimic miR-181 (Hoechst staining, ×100). **(B)** LDH release. **(C)** Caspase-3 activity. **(D)** The ratio of BAX/Bcl-2 in gastric mucosal epithelial cells. Data are expressed as mean ± standard error (*n* = 3). #*p* < 0.05 vs. MicroRNAs mimic, *p < 0.05 vs. Negative Control.

To further confirm the role of exogenous glutamate in mimic-induced gastric injury, we transfected GES-1 cells with the mimics and pretreated the cells with glutamate. The glutamate supplementation decreased the cell death (Figures 5A, 6A, 7A) and LDH leakage (Figures 5B, 6B, 7B) and promoted caspase-3 activity (Figures 5C, 6C, 7C). Western blotting showed that the three mimics inhibited xCT activity and increased the expression of apoptosis-related proteins (Figures 5D, 6D, 7D). These results further confirm that the miRNA/glutamate pathway may be involved in cold stress-induced ulcers.

## Discussion

Stress ulcer refers to acute gastrointestinal mucous erosion and ulcers caused by various stress states, such as severe trauma, critical disease, and severe psychological disease (Hong et al., 2015). In severe cases, a stress ulcer can be complicated by gastrointestinal bleeding or perforation, which can aggravate the severity of the original disease and increase the fatality rate. Epidemiological data show that the incidence of stress ulcers in intensive care unit patients is as high as 85–100%, and the mortality of stress ulcer bleeding is almost 46% (Shears et al., 2016). Stress-induced gastric ulcer models have been established in a variety of ways, including exhaustion sports, water sports exhaustion, seawater, and bound and cold-bound models (Singh et al., 2012). This study involved gastric ulcers induced in rats that were restrained in a cold environment. Visible mucous bleeding and pathomorphological changes, such as loosely arranged epithelium structure, swollen glands, and cell apoptosis, were evident. Epithelial cell apoptosis is not only an indicator of gastric mucous injury but is also an important factor that aggravates the injury. Previous studies have shown that aspirin and *H. pylori* can induce apoptosis in gastric mucous cells (Du et al., 2014). The present study confirmed the previous observation that cold stress can induce gastric cell apoptosis. We observed that the ratio of the apoptosis-related genes BAX/Bcl-2 increased with an increased caspase-3 activity in gastric tissues. However, the mechanism underlying cold stress-induced apoptosis is not fully understood. Previous studies have reported that *H. pylori* induces apoptosis and increases the production of inflammatory cytokines (Konturek et al., 1999; Konstantopoulos et al., 2001; Bartchewsky et al., 2010). Cold stress induces gastric injury, accompanied by an inflammatory reaction. Cold stress likely induces cell apoptosis secondary to an inflammatory response. In contrast, increased sympathetic activity and catecholamines induce oxidative stress and apoptosis (Ketchesin et al., 2017). We found that cold stress induced changes in the expression of certain miRNAs that play an important role in regulating apoptosis.

miRNAs are involved in regulating the physiological and pathological processes of cell proliferation, regeneration, apoptosis, and damage repair. Recent studies have found that miRNAs are important regulators of the stress response. For example, in mice with chronic mental stress, stress significantly increased the expression of miR-193-5p, miR-204, miR-29c, miR-30a, miR-30c, miR-32, miR-375, miR-532–3p, and miR-698 in sperm (Rodgers et al., 2013). The expression of miR-322*, miR-324,miR-463*, miR-674*, miR-142-5p, miR-19b, miR-1928, miR-223-3p and miR-421-3p was significantly increased in the serum of rats during traumatic stress (Balakathiresan et al., 2014). Animal experiments using deep sequencing have shown that multiple freeze-responsive miRNAs, including miR-151, miR-425, miR-92a, miR-98, and miR-328, are significantly lower in sera of rats treated with cold exposure (Guo et al., 2016). Based on the key role of miRNAs in mediating cold stress, we speculated that cold stress-induced gastric mucosa injury may also be associated with certain miRNA expression effects. In this study, we identified 22 microRNAs related to xCT using TargetScan software. We found significantly upregulated expression of miR-142-5p, miR-142-3p, miR-143-5p, miR-144-5p, miR-152-5p, and miR-181a in gastric mucosal tissue induced by cold stress. Of these, the basic expression of miR-143, miR-152, and miR-181 was relatively high. To further confirm the regulatory effect of miRNA on the apoptosis of gastric mucous induced by cold stress, we observed the effects of the miR-143, miR-152, and miR-181 mimic on epithelial cell apoptosis. All three mimics could induce GES-1 gastric mucous epithelial cell injury and apoptosis. Expression of miR-223, miR-155, miR-204, miR-181, and miR-92a in GES-1 cells is altered in *H. pylori* infection (Vasapolli et al., 2021). In ethanol-induced gastric mucous injury, miR-145 was upregulated, while miR-17, miR-19a, miR-21, miR-181a, and miR-200c were downregulated (Luo et al., 2013). These findings indicate that the miRNA pathway involves different factors, such as ethanol, *H. pylori*, and cold stress, that induce apoptosis of gastric mucous. miRNAs regulate apoptosis by influencing the apoptosis-related genes Bcl-2 and BAX. In this study, cell apoptosis induced by cold stress in rats was related to upregulated miR-143, miR-152, and miR-181, and the BAX/Bcl-2 ratio was significantly increased. *In vitro* experiments showed that miR-143, miR-152, and miR-181 mimics induced apoptosis and increased the ratio of BAX/Bcl-2.

As mentioned above, the pathological process of stress ulcers involves a variety of endogenous active substances, such as nitric oxide, calcitonin gene-related peptides, and other endogenous active substances (Duan et al., 2004; Wang et al., 2008). Glutamate, as an extracellular signal mediator in peripheral tissues via autocrine and/or paracrine signaling (Du et al., 2016), may play a protective role in gastric mucosa injury from acute aspirin irritation and cold irritation (Chen et al., 2001). For example, glutamate receptor activation can induce an inflammatory response and aggravate liver damage (Roman-Ramos et al., 2011). High concentrations of glutamate can induce inflammation and promote lung injury (Ma et al., 2014). Glutamate concentration is regulated by transporters. In humans, there are five subtypes of glutamate transporters (EAAT1–5) which are driven by energy derived from ion gradients. Another transporter for glutamate is vesicular transport xCT. Interestingly, glutamate has a protective role in damage repair by regulating oxidative reactions and immune responses in the stomach (Dixit et al., 2014). Previous studies in our laboratory have shown that the mechanisms of aspirin and *H. pylori*-induced gastric injury in rats are related to the glutamate pathway (Du et al., 2014), we found that decreased xCT activity in aspirin-and Helicobacter pylori-induced gastric injury in rat. In this study, we also studied the changes of xCT activity induced by cold stress in rats, we also found decreased xCT activity and glutamate release in cold-stressed rats. But we cannot deny the role of other subtypes of glutamate transporters, which may need further study. Furthermore, we observed a protective effect of exogenous glutamate against gastric mucosal injury. These results suggest that a reduction in xCT activity may be involved in gastric ulcers induced by different factors. Furthermore, we demonstrated that the decreased xCT may be secondary to the upregulation of some miRNAs in the cold-stressed model, such as miR-143, miR-152, and miR-181. Previous work has demonstrated that miR-30b and miR-27a negatively regulate xCT in *H. pylori*-induced gastric injury model Du et al. (2020).

In conclusion, the decreased level of glutamate by reduction of xCT activity plays an important role in gastric mucosal injury induced by cold stress, and cold stress reduces the protective effect of the glutamate pathway on gastric mucous via upregulated miRNAs. Exogenous glutamate can significantly reduce gastric mucosal injury induced by cold stress, providing an experimental basis for further research and development.

## Data Availability

The original contributions presented in the study are included in the article/Supplementary Materials, further inquiries can be directed to the corresponding authors.
